# FAMILY INVOLVEMENT AND SYMPTOM BURDEN IN NURSING HOME RESIDENTS WITH COGNITIVE IMPAIRMENT

**DOI:** 10.1093/geroni/igae098.1894

**Published:** 2024-12-31

**Authors:** Gretchen Tucker, John Cagle, Timothy Stump, Wanzhu Tu, Peiyuan Zhang, Alex Floyd, Mary Ersek, Kathleen Unroe

**Affiliations:** University of Maryland, Baltimore, Baltimore, Maryland, United States; University of Maryland Baltimore, Baltimore, Maryland, United States; Indiana University Bloomington, Bloomington, Indiana, United States; Indiana University Indianapolis, Indianapolis, Indiana, United States; University of Maryland Baltimore, Baltimore, Maryland, United States; Indiana University School of Medicine, Indianapolis, Indiana, United States; University of Pennsylvania, Philadelphia, Pennsylvania, United States; Indiana University, Indianapolis, Indiana, United States

## Abstract

Research suggests that family involvement improves the quality of life of nursing home residents. Using baseline data from an on-going multisite clinical trial (UPLIFT-AD), we examined the association between family involvement and staff-reported accounts of resident symptom burden. Symptom burden was measured as a composite of frequency and intensity of symptoms, using items from the End-of-Life Dementia (EOLD) scale. Higher scores indicate greater burden. Family involvement was measured by the family-reported average weekly in-person visit frequency over the past month (range 0-7 days). Data on 198 residents were collected, 61.9% of whom were in Indiana, with the remainder (38.1%) in Maryland. Of the 198 residents, 60.3% were female. The Brief Interview for Mental Status score, which indicates cognitive impairment level, was 6.5 (SD=3.9). Most family members were an adult child (58.7%), 7.9% were spouses, and 38.8% were “other.” On average, spouses visited 5 days a week, children 2 days a week, and other family members 1.5 days a week. In 53.5% of cases, the reporting staff member knew the resident for >1 year. Linear regression analysis was used to examine the associations between the frequency of in-person family visits and EOLD scores while adjusting for resident demographics, mental status, family relationship to the resident, and facility location. Visit frequency was associated with higher EOLD scores (B=0.18, p=.037). Findings suggest family involvement may sensitize nursing home staff to the presence of a resident’s burdensome symptoms, which may become more discernible to families with increased involvement.

